# Spatiotemporal trends in leptospirosis in the Philippines: a 10-year national surveillance analysis in a One Health and climate-related context

**DOI:** 10.3389/fpubh.2026.1871644

**Published:** 2026-10-05

**Authors:** Khristine Sandoval, Lanieleen Jerah Mae Arroyo, Nico Bravo, Allyana Villa, Christopher Allan Reballos

**Affiliations:** National University, Pasay City, Philippines

**Keywords:** epidemiology, leptospirosis, Philippines, spatial trends, temporal trends

## Abstract

**Background:**

Leptospirosis remains an important zoonotic disease in the Philippines, yet its long-term national and regional epidemiological patterns and implications for surveillance remain insufficiently characterized. This study aimed to (1) characterize temporal and spatial trends in reported human leptospirosis incidence in the Philippines from 2015 to 2024; (2) situate Philippine trends within the global epidemiological context; (3) interpret national and regional patterns through a One Health-informed perspective; and (4) examine their implications for surveillance and public health policy.

**Methods:**

A retrospective analysis of reported human leptospirosis cases and population data from 2015 to 2024 was conducted across Philippine regions. Incidence rates were calculated and temporal and regional variations were assessed using negative binomial regression with a log link and a maximum-likelihood-estimated dispersion parameter, using an offset for population. Candidate models (Poisson, negative binomial with fixed dispersion, negative binomial with estimated dispersion) were compared using deviance and Pearson dispersion ratios, the Akaike Information Criterion (AIC), and the Bayesian Information Criterion (BIC). The observed epidemiological patterns were interpreted in relation to established environmental and reservoir-related mechanisms reported in previous literature, without directly modeling animal, rainfall, or other hydrometeorological variables.

**Results:**

A negative binomial regression model with an estimated dispersion parameter (*k* = 0.29) provided the best fit for Philippine leptospirosis data from 2015 to 2024 (AIC = 1891.82; BIC = 1976.48), outperforming both the Poisson model and a negative binomial model with fixed dispersion. Adjusting for region, national incidence demonstrated significant temporal variation (*p* < 0.001). Incidence in 2015, 2016, 2017, 2019, 2020 and 2021 was significantly lower than 2024 (the reference year), while 2018, 2022 and 2023 did not differ significantly from 2024. Regional variation was also significant (*p* < 0.001), with the Bangsamoro Autonomous Region in Muslim Mindanao (BARMM), the region with the lowest observed incidence, serving as the reference category. Fifteen of sixteen comparator regions showed significantly higher adjusted incidence than BARMM (adjusted IRR ranging from approximately 5.7 in Northern Mindanao to approximately 38.0 in Western Visayas, *p* < 0.001). Only SOCCSKSARGEN did not differ significantly from BARMM (aIRR = 1.45, *p* = 0.177).

**Conclusion:**

Reported human leptospirosis incidence in the Philippines exhibited significant temporal and spatial heterogeneity from 2015 to 2024, with a sustained higher national incidence beginning in 2022 and distinct regional risk profiles. These findings are consistent with the substantial burden of leptospirosis reported globally in tropical and subtropical settings and support a One Health-informed interpretation of the disease within the human–animal–environment interface. Environmental, hydrometeorological, and reservoir-related mechanisms may provide plausible contextual explanations based on previous evidence but were not directly evaluated in this study. The findings support regionally targeted surveillance and interventions, strengthened multisectoral coordination, and future integration of human, animal, environmental, and hydrometeorological data to improve leptospirosis risk assessment and early-warning capacity.

## Introduction

Leptospirosis is a globally distributed zoonotic disease caused by pathogenic *Leptospira* species, representing a critical intersection of human, animal, and environmental health. Humans typically become infected through direct or indirect contact with the urine of infected reservoir hosts most notably rodents or through environmental exposure to contaminated soil and water (1). Flooding is a major risk factor, as it facilitates the dispersal of *Leptospira* into the environment, thereby increasing the likelihood of human contact. Consequently, outbreaks are frequently associated with extreme weather events, including heavy rainfall and monsoonal flooding (2, 3).

The Philippines is particularly vulnerable to leptospirosis due to its tropical climate, susceptibility to frequent typhoons, and widespread exposure to periodic flooding (4). These environmental hazards intersect rapid urbanization, high population density, inadequate drainage, poor waste management, and the prevalence of animal reservoirs to create conditions highly favorable for transmission. Empirical evidence from Metro Manila demonstrates a robust association between rainfall and subsequent leptospirosis incidence (5). Research indicates that disease risk peaks approximately 2 weeks after heavy rainfall, with flooding acting as a significant mediator in this relationship (6). This dynamic was clearly illustrated during the 2009 Manila floods, where a typhoon-triggered disaster led to a massive surge in cases, highlighting how extreme weather can rapidly amplify transmission pathways and overwhelm public health systems (7).

These relationships are increasingly urgent in the context of a changing climate. Climate variability specifically shifts in precipitation patterns, and the increasing frequency of extreme weather events alters the environmental conditions that dictate *Leptospira* transmission. While rainfall and flooding facilitate the dispersal of *Leptospira* in contaminated environments, disruption to critical infrastructure (water, sanitation, and housing) further increases human vulnerability. Importantly, the relationship between climatic conditions and leptospirosis is shaped by interacting social, environmental, and ecological factors. Disease risk emerges from complex, synergistic interactions among climatic drivers, environmental contamination, reservoir dynamics, human behavior, and socioeconomic status.

The One Health framework provides an essential approach for navigating these interconnected processes by recognizing that human health is inextricably linked to animal welfare and the stability of shared ecosystems. In the leptospirosis cycle, pathogens circulate within reservoir populations, while human transmission is facilitated by environmental contamination and behavioral interactions with water or soil (8). Anthropogenic modifications such as changes in land use, urban expansion, and shifts in waste management directly influence these transmission pathways (9). An integrated, multisectoral perspective is therefore necessary to move beyond clinical management and identify preventive opportunities across human, animal, and environmental health sectors (10).

Long-term surveillance is vital for identifying epidemiological trends that might otherwise be obscured by the volatility of individual outbreaks. By examining a decade of data, researchers can discern temporal trends. Seasonal patterns and geographic variations may correspond with climatic and environmental conditions. Such analyses establish an epidemiological foundation for determining whether observed disease patterns correlate with known climatic risks, ultimately helping to identify high-risk populations and locations requiring strengthened preparedness.

Thus, the objectives of this study were therefore to:

(1) Characterize long-term temporal and spatial trends in leptospirosis incidence in the Philippines from 2015 to 2024;
(2) Situate Philippine leptospirosis trends within a global comparative context;
(3) To analyze the 10-year national and regional epidemiological trends of human leptospirosis, where the findings were interpreted through a One Health-informed framework, considering established environmental and reservoir-related mechanisms reported in previous studies.
(4) Examine policy implications of the observed temporal, and spatial patterns of leptospirosis incidence in the Philippines.

## Methods

### Study design

This study employed a retrospective, ecological, quantitative research design to examine the temporal, spatial, and climate-associated patterns of leptospirosis in the Philippines from 2015 to 2024. As an ecological study, the unit of analysis is the population of each administrative region and the nation, rather than individual patients, making the design suitable for characterizing population-level disease burden rather than causal inference. Secondary surveillance data were obtained from the Department of Health’s (DOH) Philippine Integrated Disease Surveillance and Response (PIDSR) system. Annual regional case counts were cross-referenced with population denominators from the Philippine Statistics Authority (PSA) to compute standardized incidence rates per 100,000 population. The study integrated a descriptive-comparative component analyzing incidence trends across seventeen administrative regions over the 10-year period with an inferential-analytical component using generalized linear models (GLM) to determine the statistical significance of regional and yearly variations. This dual longitudinal-spatial approach allowed for a comprehensive assessment of the disease burden and its association with hydrometeorological drivers within a single analytic framework.

### Data acquisition

Leptospirosis case data (2015–2024) were sourced from the DOH Weekly.

Epidemiological Reports and the PSA. Population denominators were derived from the 2015, 2020, and 2024 PSA Census of Population. For intervening years (2016–2019 and 2021–2023), population values were calculated using geometric interpolation and extrapolation, consistent with standard postcensal estimation methods. To account for regional boundary changes specifically the 2024 reconstitution of the Negros Island Region provincial-level data for Negros Oriental and Siquijor were used to restate historical figures for Western and Central Visayas on a boundary-consistent basis. All reported leptospirosis cases and population data were compiled, harmonized, and cross validated into a unified dataset for temporal, spatial, comparative, and One Health informed analysis. This study relied exclusively on secondary government sources; no primary data collection, laboratory testing, or direct patient contact was involved.

### Case definitions

Leptospirosis cases were classified according to the PIDSR system, which aligns with World Health Organization (WHO) standards.

*Suspect Case*: A patient presenting with acute fever and at least two compatible symptoms (e.g., myalgia, headache, conjunctival suffusion, jaundice, or oliguria) with a history of exposure to potential infection sources (floodwater, contaminated soil, or animal reservoirs) within ten days of symptom onset.

*Probable Case*: A suspect case presenting with warning signs of severe disease (e.g., hemorrhage, jaundice, renal impairment, or respiratory distress) or having an epidemiological link to a confirmed case but lacking laboratory confirmation.

*Confirmed Case*: A suspect or probable case with laboratory verification via microscopic agglutination test (MAT) seroconversion, a fourfold rise in antibody titer, PCR detection, or culture isolation.

The counts analyzed represent the aggregate of suspect, probable, and confirmed cases as reported by health facilities. This reliance on facility-level classification is a noted limitation, particularly given evidence of diagnostic misclassification, where many confirmed cases are initially misidentified as other febrile illnesses suggesting that reported figures may underestimate the true population burden.

### Inclusion/exclusion criteria

The study included all suspect, probable, and confirmed cases officially reported through the PIDSR system across the seventeen administrative regions from January 2015 to December 2024. Only years with finalized, officially released case counts were included; 2025 data were excluded from inferential models due to their year-to-date nature. Regional data were included only where reliable population denominators could be established. Records lacking clear regional attribution, duplicates, or entries inconsistent with national aggregates were excluded. Individual-level clinical data, patient-identifiable information, and primary environmental or animal-sampling data were explicitly excluded from this population-level analysis.

### Data cleaning processes

The dataset underwent a systematic verification process to ensure internal consistency between regional and national figures. Discrepancies between regional sums and national aggregates were traced back to original DOH reports and reconciled where possible. In-text narrative figures were cross-referenced against tabulated data to resolve transcription errors. Boundary restatements for the 2024 Negros Island Region were documented to ensure valid longitudinal comparisons. No region-year observations were excluded from the region/year negative binomial model. All 17 regions had complete case count and population data for all 10 years (2015–2024). All corrections and exclusions were logged to maintain a transparent, reproducible audit trail.

### Statistical analysis

Analyses were performed using IBM SPSS Statistics (version 23). The unit of analysis was the region-year observation, yielding 170 observations across 17 administrative regions and 10 years (2015–2024). Descriptive statistics including incidence rates, means, and percentage changes were computed to characterize temporal and spatial distributions. A single generalized linear model was fitted with annual regional case count as the dependent variable, region (17 levels) and year (10 levels) entered simultaneously as categorical fixed factors, and the natural logarithm of population as an offset: log(E[cases]) = β₀ + region + year + ln(population). A Poisson distribution with log link was fitted first; overdispersion was assessed via the ratio of the deviance and Pearson chisquare statistics to their degrees of freedom, both of which substantially exceeded 1 (Deviance/df = 82.044; Pearson *χ*^2^/df = 101.752), indicating that the Poisson assumption of equidispersion was violated. The model was therefore refitted using a negative binomial distribution with log link, with the ancillary (dispersion) parameter estimated from the data rather than fixed (estimated value = 0.292, SE = 0.0326). A Type III Wald chisquare test confirmed that both regions (*χ*^2^ = 451.932, df = 16, *p* < 0.001) and year (*χ*^2^ = 155.784, df = 9, *p* < 0.001) contributed significantly to model fit. No region-year observations were excluded from this analysis. Adjusted the incidence rate rates (IRRs) with 95% Wald confidence intervals were derived from this single model: Table 1 reports the year-level IRRs, adjusted for region, using 2024 as the reference year; Table 2 reports the region-level IRRs, adjusted for year, using BARMM as the reference region. A significance threshold of *p* < 0.05 was applied throughout.

**Table 1:** National leptospirosis incidence rate (per 100, 000 population) and adjusted incidence rate ratios by year, 2015–2024.

Year	Observed rate	Adjusted IRRs	95% CI	*p*-value	Interpretation
2015	1.41	0.267	0.182–0.392	<0.001	~73% lower than 2024 (significant)
2016	1.75	0.274	0.187–0.402	<0.001	~73% lower than 2024 (significant)
2017	2.99	0.551	0.377–0.806	0.002	~45% lower than 2024 (significant)
2018	5.23	0.740	0.507–1.081	0.119	No significant difference from 2024
2019	3.29	0.463	0.317–0.676	<0.001	~54% lower than 2024 (significant)
2020	1.87	0.276	0.188–0.404	<0.001	~72% lower than 2024 (significant)
2021	1.89	0.310	0.212–0.455	<0.001	~69% lower than 2024
2022	4.24	1.222	0.834–1.790	0.304	No significant difference from 2024
2023	3.76	0.854	0.589–1.238	0.404	No significant difference from 2024
2024	4.45	1.000 (refrerence)	–	–	–

^1^Negative Binomial Regression (case count = region + year + offset [ln population]); IRRs represent the year effect adjusted for region; reference year = 2024; *N* = 170).

**Table 2:** Regional leptospirosis incidence rate (per 100,000 population), 2015–2024, and adjusted incidence rate ratios^1^.

Region	2015 rate	2024 rate	Mean rate (2015–2024)	Adjusted IRRs	95% CI	*p*	Interpretation
National Capital Region (NCR)	2.17	10.57	6.78	29.99	17.82–50.47	<0.001	~30× higher than BARMM (significant)
Cordillera Administrative Region (CAR)	1.16	11.83	5.47	22.86	13.56–38.53	<0.001	~23× higher than BARMM (significant)
Ilocos (I)	2.92	6.36	5.48	25.85	15.33–43.59	<0.001	~26× higher than BARMM (significant)
Cagayan Valley (II)	1.42	14.82	7.54	31.27	18.59–52.61	<0.001	~31× higher than BARMM (significant)
Central Luzon (III)	2.96	4.77	3.12	15.08	8.96–25.37	<0.001	~15× higher than BARMM (significant)
CALABARZON (IV-A)	0.38	0.50	1.33	6.49	3.83–10.98	<0.001	~6.5× higher than BARMM (significant)
MIMAROPA	1.86	5.73	2.16	8.51	5.04–14.36	<0.001	~8.5× higher than BARMM (significant)
Bicol Region (V)	0.98	11.38	3.81	14.98	8.92–25.15	<0.001	~15× higher than BARMM (significant)
Western Visayas (VI)	4.36	3.39	7.73	37.99	22.50–64.16	<0.001	~38× higher than BARMM (significant)
Central Visayas (VII)	0.57	1.58	1.61	7.14	4.22–12.09	<0.001	~7× higher than BARMM (significant)
Eastern Visayas (VIII)	1.01	3.35	3.72	10.12	5.94–17.24	<0.001	~10× higher than BARMM (significant)
Zamboanga Peninsula (IX)	1.02	3.30	2.12	7.69	4.55–12.98	<0.001	~7.7× higher than BARMM (significant)
Northern Mindanao (X)	1.00	1.83	1.23	5.69	3.36–9.62	<0.001	~5.7× higher than BARMM (significant)
Davao Region (XI)	0.92	1.52	2.04	9.74	5.75–16.48	<0.001	~9.7× higher than BARMM (significant)
SOCCSKARGE N	0.17	1.12	0.40	1.45	0.85–2.50	0.177	No significant difference from BARMM
Caraga Administrative Region (XIII)	0.46	3.91	2.40	8.93	5.27–15.14	<0.001	~8.9× higher than BARMM (significant)
Bangsamoro Autonomous Region in Muslim Mindanao (BARMM)	0.21	0.84	0.26	1.000 (reference)	–	–	Reference

1 Negative Binomial Regression (case count = region + year + offset [ln population]); IRRs represent the region effect adjusted for year; reference region = BARMM; *N* = 170).

## Results

### Leptospirosis incidence trends in the Philippines (2015–2024)

Leptospirosis remains a significant public health challenge in the Philippines, exacerbated by the country’s tropical climate and its geographic position within the Pacific typhoon belt. The following analysis consolidates ten years of surveillance data obtained from the Department of Health (DOH) Weekly Epidemiological Reports. Table 3 summarizes the annual and regional incidence rates, providing the foundation for understanding the longitudinal and spatial distribution of the disease from 2015 to 2024 (Table 4).

**Table 3:** National trend of leptospirosis incidence rate in the Philippines and by region, From 201 to 2024.

Region		Region name		Incidence rate (cases/100,000 person-year)									
2015	2016	2017	2018	2019	2020	2021	2022	2023	2024
Philippines		1.41	1.75	2.99	5.23	3.29	1.87	1.89	4.24	3.76	4.45
NCR	National Capital Region	2.17	3.41	4.36	16.34	9.00	4.15	3.89	6.48	7.38	10.57
CAR	Cordillera Administrative Region	1.16	2.25	4.40	4.36	5.89	3.56	3.28	7.04	10.96	11.83
I	Ilocos Region	2.92	6.24	8.43	11.27	6.12	1.98	1.20	4.38	5.91	6.36
II	Cagayan Valley	1.42	1.94	5.62	5.32	8.91	6.51	4.31	14.55	11.99	14.82
III	Central Luzon	2.96	2.72	2.00	4.18	3.75	1.86	1.12	3.29	4.52	4.77
IV-A	CALABARZON	0.38	0.93	0.91	2.96	1.97	1.65	0.76	2.86	0.43	0.50
	MIMAROPA Region	1.86	1.10	0.82	1.86	0.88	0.93	0.12	3.40	4.94	5.73
V	Bicol Region	0.98	1.03	1.78	2.25	2.57	2.17	2.44	3.42	10.05	11.38
VI	Western Visayas	4.36	4.35	14.42	14.91	7.07	4.42	8.69	13.39	2.32	3.39
VII	Central Visayas	0.57	0.46	2.80	1.60	0.93	0.77	2.13	3.82	1.44	1.58
VIII	Eastern Visayas	1.01	0.38	2.52	1.89	0.66	0.20	0.57	23.56	3.04	3.35
IX	Zamboanga Peninsula	1.02	0.52	1.72	2.46	0.94	0.44	0.87	6.73	3.18	3.30
X	Northern Mindanao	1.00	1.20	1.83	1.13	0.95	0.48	0.41	1.73	1.71	1.83
XI	Davao Region	0.92	0.83	3.28	2.51	1.66	1.60	2.50	4.27	1.31	1.52
XII	SOCCSKSARGEN	0.17	0.17	0.19	0.21	0.21	0.02	0.09	0.82	1.01	1.12
XIII	Caraga	0.46	0.76	1.90	7.28	0.72	0.29	1.45	4.27	2.98	3.91
BAR MM	Bangsamoro Autonomous Region in Muslim Mindanao	0.21	0.05	0.22	0.07	0.21	0.02	0.02	0.36	0.60	0.84

**Table 4:** Goodness-of-fit comparison of poisson and negative binomial regression models for leptospirosis incidence rate by region and year, Philippines, 2015–2024.

Model	Deviance	df	Deviance/df	Pearso *n χ*^2^	Pearson *χ*^2^/df	Log-likelihood
Poisson (region + year + offset	11,814.403	144	82.044	14,652.273	101.752	−6447.560
Negative binomial, *K* fixed = 1	68.583	144	0.476	85.083	0.591	−974.616
Negative binomial, *k* estimated	179.225	143	1.253	184.926	1.293	−918.907

The data reveals significant temporal fluctuations in incidence rates. Spatially, the burden remains highly heterogeneous, with specific administrative regions consistently reporting incidence rates disproportionate to their population size. The subsequent sections detail the descriptive and inferential findings, elucidating how these regional disparities and temporal surges reflect the complex interplay between environmental drivers and existing public health infrastructure.

A comparison of the three fitted models confirms that the negative binomial specification with a freely estimated dispersion parameter provides the best-fitting representation of the leptospirosis case data. The initial Poisson model showed severe overdispersion, with both the Deviance and Pearson chi-square statistics far exceeding their degrees of freedom (Deviance/df = 82.044; Pearson *χ*^2^/df = 101.752) confirming that the equidispersion assumption of the Poisson distribution (variance equal to the mean) was strongly violated and that a Poisson model was inappropriate for this dataset. Fixing the negative binomial’s ancillary dispersion parameter at *k* = 1 corrected this problem but overcorrected it: the resulting Deviance/df (0.476) and Pearson *χ*^2^/df (0.591) fell well below 1, indicating under dispersion relative to what a fixed *k* = 1 assumes, and suggesting that this fixed value did not reflect the actual extra-variance structure of the data. When the dispersion parameter was instead estimated from the data via maximum likelihood, the model’s fit statistics moved much closer to the ideal benchmark of 1.

(Deviance/df = 1.253; Pearson *χ*^2^/df = 1.293), indicating a well-calibrated, appropriately dispersed model. This improvement is further confirmed by the information criteria: the negative binomial model with maximum likelihood-estimated dispersion achieves the lowest AIC and BIC of the three. Taking together, these diagnostics support the negative binomial model with an estimated dispersion parameter as the most appropriate and bestfitting specification for the region-year leptospirosis case data, and this is the model used to generate the adjusted incidence rate ratios reported in Tables 1, 2.

The national leptospirosis incidence rate in the Philippines exhibited significant temporal variation between 2015 and 2024. A single negative binomial regression model (case ~ region + year, offset = ln[population], *N* = 170 region-year observations) was fitted to formally test temporal variation in national leptospirosis incidence, adjusting for regional differences. The year factor contributed significantly to model fit (Type III Wald *χ*^2^ = 155.784, df = 9, *p* < 0.001), confirming that incidence varied significantly across the study period independent of regional composition. As presented in Table 1, adjusted incidence rate ratio in 2015, 2016, 2017, 2019, 2020 and 2021 were significantly lower than the 2024 reference year (aIRRs = 0.267–0.463, *p* ≤ 0.01), with 2020 showing the steepest reduction (aIRR = 0.276, 95%CI = 0.188–0.404, *p* < 0.001). The years 2015 and 2016 are ~73% lower than 2024, whereas 2017 is ~45% lower. In contrast, the year 2018, 2022 and 2023 did not differ significantly from the 2024 rate (*p* > 0.05). This indicates that incidence in these years had already reached a level statistically indistinguishable from the study’s endpoint. This pattern supports a “step-change” rather than linear interpretation of the national trend: rather than a smooth year-on-year climb, incidence appears to have shifted upward around 2018 and again after the 2019–2021 pandemic era decline. This stabilizes at a higher endemic baseline from 2022 onward.

The same model’s region factor was also a significant predictor of incidence (Type III Wald *χ*^2^ = 451.932, df = 16, *p* < 0.001), adjusting for year. Using BARMM as the region with the lowest mean incidence rate (0.26/100,000), as a reference category. Table 1 shows that the majority of administrative regions had significantly higher adjusted incident rate ratio (aIRR = 6.49–37.99, *p* < 0.001) relative to BARMM. Region VI (Western Visayas), the region with the highest raw observed rate, has also the highest adjusted IRR (aIRR = 37.99, *p* < 0.001). The aIRR is 38 times higher than the computed aIRR of BARMM. Moreover, steepest disparity was observed in NCR (aIRR = 29.99, *p* < 0.001), CAR (aIRR = 22.86, *p* < 0.001), Ilocos region (aIRR = 25.85, *p* < 0.001) and Cagayan Valley (aIRR = 31.27, *p* < 0.001), which are, respectively, ~30×, ~23×, ~26× and ~31× higher than the reference BARMM. Only Region XII (SOCCSKSARGEN) has no significant difference in aIRR with BARMM (1.45 vs. 1.00, *p* = 177).

## Discussion

### Human health impact surveillance challenges: a One Health perspective

Leptospirosis remains an important but underrecognized zoonotic disease globally. A modeling analysis estimated approximately 1.03 million cases and 58,900 deaths annually worldwide, corresponding to an estimated annual incidence of 14.77 cases per 100,000 population and mortality of 0.84 deaths per 100,000 population (11, 12). The highest estimated burdens were concentrated in tropical regions, particularly South and Southeast Asia, where environmental exposure, occupational risks, urban poverty, and limited surveillance capacity contribute to disease transmission and underestimation of the true burden (13).

Against this global background, the present analysis demonstrates substantial temporal and spatial heterogeneity in reported leptospirosis incidence in the Philippines from 2015 to 2024. National incidence increased by 216% over the study period, although the trajectory was not linear. A pronounced peak occurred in 2018, followed by a decline during 2020–2021 and a subsequent resurgence from 2022 to 2024. The pandemic associated decline should be interpreted cautiously because reductions in mobility, healthcare utilization, diagnostic testing, and routine surveillance may have affected case ascertainment during this period. Thus, changes in reported incidence may reflect a combination of true epidemiological variation and changes in surveillance intensity.

The regional patterns further demonstrate that the national burden is not geographically uniform. Five epidemiological profiles were particularly evident. The National Capital Region (NCR) represented a high-incidence persistence, in 100,000 population, increasing from 2.17 to 16.34 incidence rate from the year 2015 to 2018, and staying elevated at 10.57 incidence rate in 2024 (12, 14). NCR’s adjusted incidence was approximately 30× higher than BARMM, with an adjusted incidence rate ratio (IRR) of 29.99 (95%CI: 17.82–50.47, *p* < 0.001). In contrast, Region II—Cagayan Valley represented an emerging high-burden zone, with incidence per 100,000 population increasing from 1.42 to 14.82 from 2015 to 2024, respectively. Its adjusted incidence was approximately 31× higher than BARMM (aIRR = 31.27, 95%CI: 18.59–52.61, *p* < 0.001). The Ilocos Region (Region I) exhibited a more moderate trajectory, reaching 11.27 incidence rate in the year 2018 before declining to 6.36 in 2024. Its adjusted incidence was approximately 26× higher than BARMM (aIRR = 25.85, 95% CI: 15.33–43.59, *p* < 0.001). Cordillera Administrative Region (CAR) also demonstrated an emerging pattern, increasing from 1.16 in 2015 to 11.83 in 2024. Its adjusted incidence was approximately 23× higher than BARMM (aIRR = 22.86, 95%CI: 13.56–38.53, *p* < 0.001). Lastly, Western Visayas (Region VI), which exhibited the highest decade-long mean incidence, showed substantial year to-year volatility and an adjusted IRR of 37.99 (95%CI: 22.50–64.18, *p* < 0.001) relative to the reference region. Notably, SOCCSKSARGEN was the only region without a significantly elevated incidence relative to BARMM (aIRR = 1.45, 95% CI = 0.85–2.50, *p* = 0.177).

These contrasting trajectories suggest that Philippine leptospirosis transmission is shaped by heterogeneous local conditions rather than by a single national mechanism (15). Persistent transmission in highly urbanized areas, rapid increases in emerging regions, and episodic surges in other areas may reflect differences in population density, land use, sanitation, flood exposure, occupational activities, reservoir host ecology, healthcare access, and diagnostic capacity (5).

The observed geographic heterogeneity also highlights an important limitation of relying exclusively on reported human cases. Leptospirosis presents with nonspecific clinical manifestations and can resemble other acute febrile illnesses, complicating diagnosis. The WHO emphasizes the importance of epidemiological context and laboratory confirmation, including serology and molecular detection, in establishing confirmed cases. Recent One Health evidence similarly notes that leptospirosis is frequently misdiagnosed and consequently underestimated, while diagnostic practices vary considerably across human and animal studies (11, 16, 17).

Therefore, the national estimates presented in this study should be interpreted as reported surveillance incidence rather than the absolute incidence of infection in the population. The apparent differences between low national baselines and high regional peaks may partly reflect differences in case detection, healthcare utilization, laboratory capacity, and reporting completeness. Strengthening surveillance therefore requires not only increasing case reporting but also improving diagnostic standardization and integrating information across human, animal, and environmental health sectors (18, 19).

Despite these zones, leptospirosis remains widespread yet severely underdiagnosed. This underdiagnosis represents a failure of current integrated surveillance (20, 21). The current disparity between low national reported baselines and high localized outbreak rates is largely a product of surveillance methodology active (5, 11, 12, 22), population-based surveillance yields significantly higher morbidity data than passive hospital reporting. Through an integrated multi-sectoral approach, the absence of standardized diagnostic procedures and unified data systems creates a critical surveillance gap in public health. This fragmentation makes it difficult to determine whether fluctuations in case numbers reflect genuine disease patterns or are merely artifacts of a disconnected healthcare infrastructure (23).

### One Health-informed interpretation of epidemiological patterns

Leptospirosis is a zoonotic disease occurring at the interface of human, animal, and environmental health. Its transmission involves interactions among pathogenic *Leptospira*, reservoir hosts, environmental conditions, and human exposure. In the present study, these established relationships were considered as contextual factors in interpreting the observed temporal and spatial patterns of reported human leptospirosis incidence. Because the present analysis was based on human case and population data, animal, environmental, rainfall, and hydrometeorological variables were not directly evaluated. Therefore, these factors are discussed as plausible mechanisms supported by previous evidence rather than as statistically demonstrated determinants of the observed patterns.

In the present study, a One Health-informed perspective was used to contextualize the observed temporal and spatial patterns of reported human leptospirosis incidence. The analysis was based on reported human cases and population data; therefore, animal infection, environmental contamination, rainfall, flooding, and other hydrometeorological variables were not directly included in the dataset or statistically evaluated (24). Accordingly, the temporal and spatial patterns identified in this study should not be interpreted as demonstrating causal or statistical relationships with specific environmental, climatic, or animal-related factors.

Nevertheless, previous research has established plausible mechanisms through which environmental and reservoir-related conditions may influence leptospirosis transmission (1, 25, 26). Pathogenic *Leptospira* may be maintained in animal reservoirs and introduced into the environment through infected animal urine, creating opportunities for human exposure through contaminated soil or water. Previous Philippine studies have also reported associations between rainfall, flooding, and leptospirosis incidence (6, 27, 28). These findings provide an epidemiological and biological context for interpreting the observed patterns, but they do not constitute evidence that environmental or reservoir-related factors statistically explain the incidence patterns identified in the present analysis.

The temporal patterns observed in this study including the overall increase in reported incidence, the peak in 2018, the decline during 2020–2021, and the subsequent resurgence from 2022 to 2024 occurred within a broader context of potential demographic, environmental, healthcare, and surveillance changes. Similarly, the marked regional heterogeneity, including substantial increases in Cagayan Valley and CAR, a more moderate trajectory in Ilocos, and greater variability in Central and Western Visayas, may reflect differences in population characteristics, urbanization, land use, occupational exposure, sanitation, healthcare access, surveillance capacity, and environmental conditions. However, these factors were not directly measured in the present study and therefore cannot be identified as determinants of the observed incidence patterns (29–31).

Hydrometeorological events may provide plausible contextual explanations for some of the temporal variation observed. Previous Philippine research has documented temporal and lagged associations between rainfall, flooding, and leptospirosis, while PAGASA reports provide evidence of recurrent heavy rainfall, typhoons, and flooding during portions of the study period (4, 6). Such evidence supports the plausibility of environmental pathways in leptospirosis transmission. However, because hydrometeorological variables were not incorporated into the present analysis, these events should be regarded as contextual factors supported by previous evidence rather than statistically demonstrated drivers of the incidence patterns reported in this study.

A One Health-informed perspective further emphasizes that reported human cases represent only one component of a broader human–animal–environment interface. The regional differences identified in this study may help identify areas where integrated surveillance and further investigation of environmental and reservoir-related factors are warranted. This perspective does not imply that these factors explain the observed patterns but rather highlights the importance of considering them when developing hypotheses for future investigation and surveillance planning.

Future research should incorporate complementary data across the human, animal, and environmental domains, including animal surveillance, environmental indicators, landuse characteristics, sanitation and water-related variables, and relevant hydrometeorological information. Linking these datasets with human surveillance records would allow formal assessment of whether environmental or reservoir-related factors are associated with the temporal and spatial variation in Philippine leptospirosis incidence. Such integrated datasets could also provide a stronger evidence base for developing targeted early warning and risk-prediction approaches.

Overall, the present findings demonstrate substantial temporal and spatial heterogeneity in reported human leptospirosis incidence from 2015 to 2024. A One Health-informed perspective provides a conceptual basis for interpreting these patterns within the broader human–animal–environment interface, while maintaining a clear distinction between factors demonstrated by the present dataset and mechanisms supported by previous research. Environmental and hydrometeorological factors should therefore be presented as plausible, literature-supported contextual explanations rather than statistically demonstrated determinants of the observed incidence patterns.

### Policy implications for surveillance and governance

The temporal and spatial patterns identified in this study have direct implications for surveillance and public health governance (32, 33), the 216% increase in incidence between 2015 and 2024 reflects the limitations of routine, passive human-case reporting (6, 15). The distinct peaks in our data aligned with severe weather years like 2018, 2022, and 2024 support previous findings that meteorological and environmental data must be integrated into health surveillance systems to be effective (15, 34, 35).

The Philippine policy framework provides complementary mechanisms for addressing these challenges. Republic Act No. 11332 supports mandatory disease reporting and surveillance, including the Philippine Integrated Disease Surveillance and Response (PIDSR), while Republic Act No. 10121 provides the framework for disaster risk reduction and management. Strengthening coordination between these systems, together with environmental, veterinary, and local government sectors, could support more integrated leptospirosis preparedness.

The distinct regional profiles identified in this study further support risk-stratified surveillance and intervention. Persistent high-incidence areas such as Western Visayas may require sustained clinical and community-level preparedness, while rapidly increasing regions such as Cagayan Valley and CAR warrant strengthened surveillance and prevention. Future research should evaluate hydrometeorological and other environmental indicators as potential early-warning inputs before their predictive value is incorporated into surveillance systems.

## Conclusion and recommendation

This study characterized the temporal and spatial patterns of reported human leptospirosis incidence in the Philippines from 2015 to 2024, demonstrated their relevance within the broader global epidemiology of leptospirosis, interpreted the observed patterns through a One Health-informed perspective, and identified implications for surveillance and public health policy.

First, the analysis demonstrated substantial temporal and spatial heterogeneity in reported leptospirosis incidence over the ten-year period. National incidence increased substantially between 2015 and 2024, with a pronounced peak in 2018, a decline during 2020–2021, and a subsequent resurgence from 2022 to 2024. Regional patterns were also distinct, with marked increases in Cagayan Valley and CAR, a more moderate trajectory in Ilocos, and considerable temporal variability in Central and Western Visayas. These findings demonstrate that leptospirosis burden is not uniformly distributed across the country and that national surveillance data can reveal distinct regional epidemiological profiles.

Second, the Philippine pattern is consistent with the global recognition of leptospirosis as an important zoonotic disease, particularly in tropical and subtropical regions. However, the magnitude and distribution of reported incidence in the Philippines should be interpreted within the country’s specific demographic, geographic, socioeconomic, and health-system context. Differences in surveillance systems, diagnostic capacity, case ascertainment, and reporting practices should also be considered when comparing Philippine trends with international estimates.

Third, the observed epidemiological patterns were interpreted through a One Health informed perspective, recognizing that leptospirosis occurs at the human–animal–environment interface. Previous studies support the plausibility of environmental, hydrometeorological, and reservoir-related mechanisms in leptospirosis transmission. However, these factors were not directly measured in the present study. Therefore, they should be regarded as literature-supported contextual explanations rather than statistically demonstrated determinants of the temporal and spatial patterns identified in this analysis. The findings highlight the value of future integrated surveillance incorporating human, animal, and environmental data.

Fourth, the temporal and regional patterns have important implications for surveillance and public health policy. The persistence of high incidence in some regions and the rapid increases observed in others support a more geographically differentiated approach to surveillance and preparedness. Existing national mechanisms for disease surveillance and disaster risk reduction provide opportunities to strengthen coordination between epidemiological surveillance, local health systems, environmental management, and disaster preparedness. Such integration may facilitate more timely identification of highrisk settings while avoiding assumptions about causal environmental drivers that were not evaluated in the present study.

*Based on the findings and their limitations, the following recommendations are proposed*:

1. Strengthen national and regional epidemiological surveillance. Maintain and improve timely reporting of human leptospirosis cases and population-based incidence estimates, with particular attention to regions demonstrating persistent high incidence or substantial increases over time. Regional surveillance profiles should be regularly reviewed to identify emerging changes in disease burden.
2. Develop region-specific prevention and preparedness strategies. Interventions should reflect the distinct epidemiological profiles identified in this study. Persistent high-incidence areas such as NCR may require sustained clinical preparedness and community-level prevention, while rapidly increasing regions such as Cagayan Valley and CAR warrant strengthened surveillance and early intervention. Regions with substantial temporal variability, such as Western Visayas, may benefit from flexible outbreak preparedness mechanisms.
3. Strengthen one health-informed surveillance and research. Future surveillance should progressively incorporate relevant human, animal, and environmental information. Animal-reservoir surveillance, environmental monitoring, land-use characteristics, sanitation and water-related indicators, and other relevant ecological variables could complement human case surveillance and provide a more comprehensive understanding of transmission patterns.
4. Evaluate hydrometeorological indicators for future early-warning systems. Although hydrometeorological variables were not analyzed in the present study, previous Philippine research and PAGASA reports provide plausible contextual evidence for their relevance to leptospirosis transmission. Future studies should formally assess rainfall, flooding, and other relevant hydrometeorological indicators alongside human surveillance data before their use as predictive inputs in an early warning system.
5. Strengthen multisectoral policy coordination. The complementary mandates of Republic Act No. 11332 on disease surveillance and reporting and Republic Act No. 10121 on disaster risk reduction and management can provide a policy basis for closer coordination among public health, disaster risk reduction, environmental, veterinary, and local government sectors. Such coordination should support evidence-based preparedness while maintaining a clear distinction between observed epidemiological patterns and hypothesized environmental or reservoir-related mechanisms.

Overall, the study demonstrates that Philippine leptospirosis exhibits substantial and geographically heterogeneous temporal patterns that warrant sustained and regionally responsive surveillance. The findings support a One Health-informed approach to future research and policy development, while emphasizing that environmental, hydrometeorological, and animal-related factors require direct investigation before being considered statistically established drivers of leptospirosis incidence.

## Data Availability

The datasets presented in this study can be found in online repositories. The names of the repository/repositories and accession number(s) can be found in the article/supplementary material.
